# Is Osteoporosis Infectious?

**Published:** 1892-04

**Authors:** J. C. McNeil


### IS OSTEOPOROSIS INFECTIOUS?

By J. C. McNeil, V. M. D.

Possibly no disease so frequently met with by the veterinarian has such meagre literature on the subject as this very important one of Osteoporosis. This disease, which a few years ago was comparatively unknown to us, has become quite prevalent, and I have good reasons to believe that the number of cases are multiplying very rapidly, and now is the time for the profession to investigate the cause of this great increase. Many theories have been advanced as to the cause of this strange disease, but I don't believe any of them can be proven to be founded on scientific or clinical facts. It is not the object of this paper to describe a disease of which you are all acquainted, and I will leave the symptoms, alterations, prognosis and treatment severely alone and in the hands of more able writers, and deal entirely with the etyology.

In no literature on Osteoporosis have I ever noted "infection" as a cause, even hinted at, and in my affirming that it is, I believe I stand pre-eminently alone. While I may be wrong, I think I have had excellent reasons for forming such belief, and all I can hope for is that this article may furnish food for reflection and that some good may be the ultimate result.

For the past two years I have been Veterinarian for five street railway companies, and made at least one trip a day to each of the several stables. At the stables of the Long Line and Rebecca Street Company, there was 220 head of stock; at Short Line stables, about 100 head; at the Union Line stables, 165 head; at Troy Hill stables, 75 head, and the Spring-Garden Avenue line has about 127 head. In my capacity as Veterinarian I had a valuable opportunity to watch the progress of this disease, which was very prevalent in some of the stables, while in others a case was never known to exist. The stock used on these various lines were of the same class, size and build; the kind of work precisely the same; they were all fed the same kind of food, the same amount, and in the same manner; and as for hygienic surroundings, I am compelled to say that the best stables had the greatest number of cases, and in two of three stables where one would naturally think to find such a disease, as they were damp, foul and poorly constructed, in these two stables there never was a case of Big-head. I will also say right here that in looking up the locality from which these affected horses came, I found made very little or no difference, as animals that were raised on a clay or sandy soil were as frequently infected as those coming from a lime-stone country, which latter soil some authorities give as a cause. Neither did I find sex, age or temperament important factors, because it made very little or no difference whether the animals were male or female, old or young, and as for temperament, I believe the majority was of the sanguinary.

At the stable which housed the 220 head of stock used on the Rebecca Street Line, and the Long Line, during a period of two years, I had forty-seven well-marked cases of Osteoporosis, and a few more very suspicious cases. This stable was an ideal stable as far as hygiene is concerned, as it was substantially built, well ventilated, good light, dry, and excellent system of drainage, and the management very good.

At the Short Line stables, where only 100 head were kept, I found during the same space of time 26 well-marked cases; and here again we have a first-class stable, being dry, well ventilated and clean, and the management good. At this stable you will see there is a greater proportion of cases than at any of the others, and at this stable the horses had the easiest work of any, as the run was shorter, and the time to make the trip is slower than any of these lines.

The Union Line stables were a miserable affair, being built on low and swampy ground; the stable was always wet and foul, and while there was always a great deal of work for me to do here, much of which was caused by the bad condition of the place, still I must say that I only had two well-marked cases and two suspicious cases, which latter had been disposed of to "dealers," and I could not trace them further. Strange to say, these few cases occurred in the last six months of the stable's existence, as this road has adopted electricity as a motive power. During this space of time the company did not think it justified them to buy much stock, and often in a "pinch" horses were brought from the Long Line stables to that of the Union Line to help them out, and these *extra* horses from the Long Line stables might have carried the infection; at any rate, there was no Osteoporosis previous to this interchanging of stock from one stable to the other.

At the Troy Hill Line and Spring-Garden Avenue stables during this same period of time, a case of Osteoporosis was never known of. Now here are two stables where the stock was called on to do the same kind of work; they were the same class of stock as the others, fed the same kind of food and in the same manner as at the other barns, and the only difference was in their hygienic surroundings, which, by the way, is very much inferior to that of the first two barns described.

I could cite several other instances met with in my general practice, where I have observed two or more cases occurring in "private" stables, which, if described, would still further strengthen my argument that Osteoporosis should be classed among the infectious diseases.

Prof. Williams, in his *Principles and Practice of Veterinary Surgery*, gives the report of Prof. Varnell, which latter gentleman was the first to describe the disease in 1860. Prof. Varnell reports the finding of no fewer than six cases of this strange malady on one farm, and that these horses had been, all but one, bred on the same farm, and were the offspring of different mares and different sires. The facts gained by Prof. Varnell may be briefly stated as follows: They had been fed on the same food as other horses on the same farm that had never shown any symptoms of the disease. The owner had been in the habit of feeding his horses in the same way for years without any previous ill-effects. They drank the same kind of water as the other stock. Prof. Varnell examined the food they were getting and pronounced it good. Another fact that he mentions is most interesting, namely, that this same gentleman had another farm only a short distance from the farm where this disease existed, and on that farm not a single instance occurred, although the horses on this farm were bred from the same parents and partook of the same kind of food as on the first-described farm. This report of Prof. Varnell's positively does not weaken my theory.

In conclusion, I would say that the finding of 47 cases of Osteoporosis in one stable during a period of two years, and 26 cases in another stable which only held 100 head during the same space of time, would certainly create a strong suspicion that this is an infectious disease; at any rate, I can only hope that this short article may furnish food for reflection, and that some good may be the ultimate end, and I believe the veterinarian or bacteriologist who will investigate on this line of thought will find a fertile field at his disposal, and his efforts be crowned with success.

